# The Management Perspective in Digital Health Literature: Systematic Review

**DOI:** 10.2196/37624

**Published:** 2022-11-10

**Authors:** Alfred Angerer, Johanna Stahl, Egzona Krasniqi, Stefan Banning

**Affiliations:** 1 Healthcare Management Winterthur Institute of Health Economics Zurich University of Applied Sciences Winterthur Switzerland; 2 University Hospital Zurich Zurich Switzerland; 3 Department of Health Sciences University of Applied Sciences Fulda Fulda Germany

**Keywords:** digital health, management, health care management, literature review, health technology, eHealth, data health, trend health, tech health

## Abstract

**Background:**

New digital health technologies are considered one solution to challenges in the health sector, which include rising numbers of chronic diseases and increased health spending. As digitalization in health care is still in its infancy, there are many unanswered questions about the impact of digital health on management.

**Objective:**

This paper assesses the current state of knowledge in the field of digital health from a management perspective. It highlights research gaps within this field to determine future research opportunities.

**Methods:**

A systematic review of digital health literature was conducted using 3 databases. The chosen articles (N=38) were classified according to a taxonomy developed for the purpose, and research gaps were identified based on the topic areas discussed.

**Results:**

The literature review revealed a slight prevalence of practical (n=21, 55%) over theoretical (n=17, 45%) approaches. Most of the papers (n=23, 61%) deal with information technology (IT) and are, therefore, focused more on technology and less on management. The research question in most of the papers (n=31, 82%) deals with the creation of concepts, and very few (n=4, 11%) evaluate or even question existing solutions. Most consider the main reason for digitalization to be the optimization of operational processes (n=26, 68%), and 42% (n=16) deal with new business models. The topic area discussed most frequently was found to be eHealth (n=30, 79%). By contrast, the field of tech health with topics such as sensors receives the least attention (n=3, 8%), despite its significant potential for health care processes and strategy.

**Conclusions:**

Three main research propositions were identified. First, research into digital health innovation should not focus solely on the technology aspects but also on its implications for strategic and operational management. Second, the research community should target other domains besides eHealth. Third, we observed a lack of quantitative research on the real impact of digital health on organizations and their management. More quantitative evidence is required regarding the expected outcome and impact of the implementation of digital health solutions into our health care organizations.

## Introduction

### Background

Any list of the significant trends that influence the way we manage our organizations will certainly include digitalization. It is not surprising, therefore, that digital health is a major area of interest in the health care sector. Public health literature, popular media, and health care services all increasingly focus on this topic [1]. At the same time, a uniform definition of the term ‘digital health’ has yet to be established [2]. Furthermore, when discussing digitalization in the health care sector, the terms Medicine 4.0, Health 2.0, and ‘connected health’ are used alongside and sometimes interchangeably with digital health.

It should be emphasized that digital health has the potential to radically change the strategy, operations, and culture of health care providers. In particular, it can offer cost-effective, patient-centered solutions [3], which could drastically simplify access to data as well as the exchange and generation of data for the benefit of patients and health care professionals [4].

Furthermore, digital health can add value not only at the level of the overall health care sector but also to individual organizations; therefore, it also has a significant management perspective.

When discussing digital health solutions and taking advantage of the great potential associated with them, individual health care organizations and their managers are confronted with major decisions. In order to be able to continue to compete in the health care market, health care managers need guiding frameworks and sound advice from scientific sources on how to exploit the potential of digital health and how to cope with the associated changes in the best possible way. We felt it was important, as a result, to assess the impact that digital health has on the field of health care management. The reverse effect, the impact of management on digital health, is beyond the scope of this study.

### Previous Research and Research Gap

Limited information is currently available on the impact of digital health from a management perspective. In a literature review of digital transformation cases by Ivančić et al [5], only 2 out of 29 papers analyzed addressed the health care sector. A literature review by Henriette et al [6] on digital transformation identified just one paper about the health sector. Admittedly, these reviews have the following limitations: (1) they were not targeted at the health care sector specifically, and (2) they had a very narrow focus on the transformative aspect of digitization.

### Research Problem, Questions, and Methods

This study addresses the lack of health care–related data in the literature on digital transformation identified above. In particular, it discusses the results of a literature review we conducted on the impact of digital health on health care management.

To this end, we followed the recommendations of Tranfield et al [7]. In addition to a comprehensive overview of digital health in the health care sector, we wanted to incorporate a broader, ‘big picture’ view of the impact of digital health on health care management. According to Thompson et al [8], health care managers and their tasks can be defined as follows:

The profession that provides leadership and direction to organizations that deliver personal health services, and to divisions, departments, units or services within those organizations [8].

Since there are many definitions of digital health, we used the following broad definition of digital health:

Digital health is the utilization of modern information and communication technologies (ICT) in the health care sector to improve the quality, the efficiency, and the focus on patients’ needs [9].

This holistic definition includes, for example, the many existing digital devices and apps used to diagnose and treat disease, simplify the self-treatment of chronic diseases, and monitor health parameters and daily behavior patterns. The definition also encompasses completely different technologies such as software used by health care providers to optimize their daily operations and train their staff.

The objective of this systematic review was to provide health care managers with an overview of digital health literature from a management perspective and uncover any potential research gaps in this field.

## Methods

The literature review we conducted is based on the approach by Tranfield et al [7] and is depicted in Multimedia Appendix 1. In the following section, the study design is presented in more detail.

### Search Strategy

In March 2019, two researchers were tasked with searching the 3 databases ABI/Inform Global, WISO, and PubMed for studies published between 2000 and 2019. The search was limited to freely available full-text articles published in English. To take into account the management perspective of digital health, the search terms “digital,” “health,” and “manage*” were used in different combinations.

Reference lists of systematic reviews were searched for additional studies not captured by our initial systematic research. Once the search was complete, duplicates were removed, and the citations were uploaded to a secure internet-based platform.

### Selection Criteria and Data Collection

Multimedia Appendix 2 defines the specific inclusion criteria for each database and shows the documentation of the search terms with the corresponding hits per database.

Studies were included according to the following inclusion criteria: (1) studies were published between January 1, 2000, and December 31, 2019, on Web of Science Core collection; (2) studies were published between 2009 and 2019 on ABI/Inform Global or PubMed; (3) studies were published in English; (4) full-text articles were freely available; and (5) studies were relevant to our subject.

Exclusion criteria included the following: (1) a lack of thematic focus (eg, ‘health’ or ‘digital’ were not the main topic or digital health was only discussed in passing); (2) studies focusing on digitalization as a means to transforming customer experience; and (3) unpublished literature, conference abstracts, and letters or editorials. Since our goal was to provide a first and extensive overview of digital health literature from a management perspective, there were no restrictions on the type of study designs reviewed.

The 2 reviewers each selected studies for possible inclusion based on title and the content of the abstract. Studies deemed to fulfill the inclusion criteria were analyzed in full-text review. Any disagreements were discussed between the reviewers, and a third party was involved to help reach consensus if necessary. Full data extraction, including characteristics of included articles, was completed by one reviewer and verified by the second reviewer.

### Analysis Framework

To analyze the literature we had identified as relevant, we developed a taxonomy with the following 6 dimensions:

1. Research approach. Publications can derive their knowledge from real implementations or theoretical thinking. In line with Brandao de Souza [10], we classified the articles as ‘case-based’ or ‘conceptual.’ This classification is relevant since, from the distribution of both types, we can derive statements on the maturity level of the overall implementation of digital health in the health care market.
2. Research question type. Wytrzens et al [11] defined 5 basic types of research question that categorize articles according to their primary objective, as follows: description, explanation, creation or concept, evaluation or criticism, and forecasting or prediction. This differentiation permits statements on the focus and core objective of a study.
3. Management discipline. The objective of this category was to assess whether various business management disciplines are treated in a balanced way. To do so, we used the same catalog that Harvard Business Publishing [12] uses to structure their publications. Since some of the articles were also in the legal and public health fields, these two dimensions were added to the Harvard Business Publishing categorization. The dimension ‘general management’ is used for any residual topic that does not fit well into the Harvard taxonomy.
4. Reason for digitalization. Like any other organization, health care organizations should always ask themselves why they want to introduce digital health solutions into their operations. In line with the message in Simon Sinek’s popular management book, “Start With Why” [13], we tried to understand the rationale for digitalization. We differentiated between the two goals of improving operational processes and creating new business models. This is a concept used by the Massachusetts Institute of Technology Center for Digital Business [14]. Their research has shown that it is primarily the business process, business model, or customer experience that can improve overall business outcomes. For our literature research, we wanted to focus on the management aspect, so we excluded all ‘customer experience’ publications.
5. Content domain. To analyze the content of the papers chosen, we used the following 4 digital health subcategories created by Angerer et al [2]: trend health (lifestyle), eHealth (exchange of data), tech health (hardware), and data health (software). During the coding, we added a fifth additional domain that we called ‘overarching challenges,’ where the focus was on the challenges in the implementation and use of different technologies found in practice.
6. Implementation approach. To answer the question of how the health care sector implements its digitalization initiatives, the examples of practices, principles, and tools from Angerer et al [2] were taken and supplemented with new elements discovered during the screening process.

## Results

### Selection

**Figure 1:**
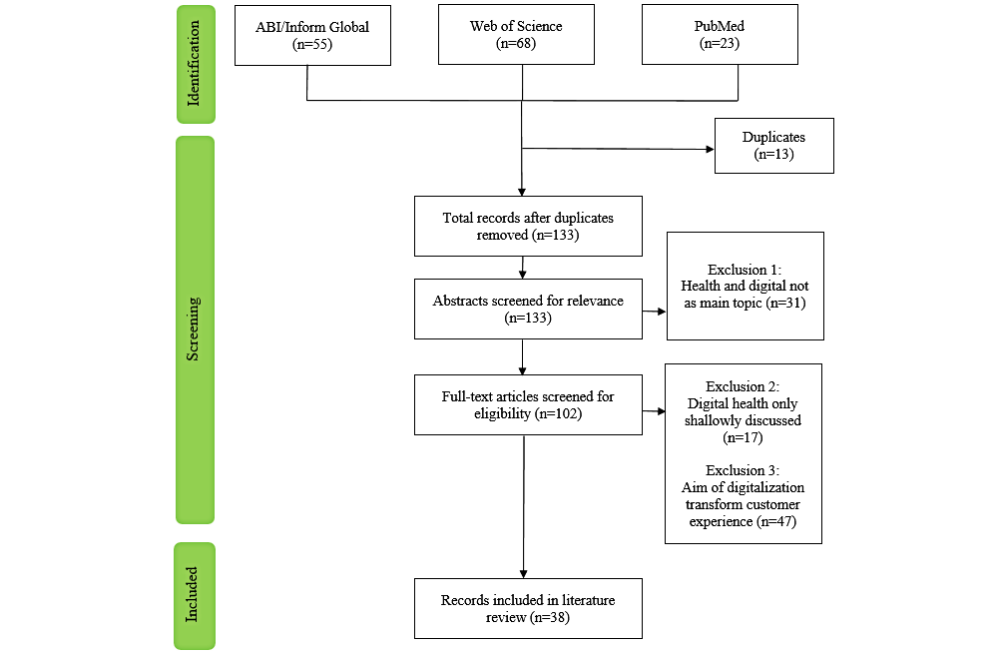
PRISMA (Preferred Reporting Items for Systematic Reviews and Meta-Analyses) flowchart of literature research procedure [15].

The search resulted in 133 unique citations, which were screened by our researchers. Based on the articles’ titles and abstracts, 31 were excluded, resulting in 102, which were subjected to a full-text screening. This process left us with 38 papers that met the inclusion criteria for our review (Figure 1). Their reference lists were searched, but no additional studies were added. Multimedia Appendix 3 shows all publications eventually included in this study.

### Analysis Based on Research Approach

Table 1 outlines the distribution of papers by research approach. Of the 38 papers included, 21 (55%) papers are case based, and 17 (45%) papers are theory based. This led us to conclude that this subject area is well balanced in terms of theory-based versus practice-based articles.

**Table 1:** Number of papers by research approach (N=38).

Research approach	Number of papers, n (%)
Case-based papers	21 (55)
Theory-based papers	17 (45)

### Analysis Based on Research Question Type

As depicted in Table 2, the objective of 31 (82%) papers was to design new digital concepts to overcome practical problems in health care. The second most pursued goal in 7 (18%) papers was to predict future health care scenarios. As frequently stated, the health care sector is still in its digital infancy; therefore, it seems highly plausible that the most popular research questions will deal with future, hypothetical situations, or concepts. We found it surprising that only 4 (11%) papers were (critical) evaluation papers, since they have one of the most vital roles of the scientific management community.

**Table 2:** Number of papers by objective of the research question (N=38)^a^.

Research question type	Number of papers, n (%)
Creation or concept	31 (82)
Forecasting or prediction	7 (18)
Description	6 (16)
Evaluation or criticism	4 (11)
Explanation	3 (8)

^a^One paper can include more than one category; therefore, the sum can be larger than 100%.

### Analysis Based on Management Discipline

As shown in Table 3, the discipline most frequently addressed in 23 (61%) papers is IT management. This finding is consistent with the findings of a literature review by Reis et al [16] on digital transformation in general and shows that IT is still the main driver of digital health (ie, technology push). From a management perspective, strategy should lead the way (ie, technology pull), yet only 4 (11%) reviewed papers addressed this underresearched topic. In a hospital setting, for example, this could imply that the IT department with its technological perspective is in the driver’s seat. Yet this can be problematic, as the digital health strategy should support the overall organizational strategy. Another indication that digital health is still primarily a result of technology push is that people-focused disciplines, such as human resources, ethics, and organizational behavior, are underrepresented.

**Table 3:** Number of papers by management discipline (N=38)^a^.

Management discipline	Number of papers, n (%)
Information technology management	23 (61)
Public health	9 (24)
General management	5 (13)
Strategy	4 (11)
Human resources management	3 (8)
Operations management	3 (8)
Entrepreneurship	2 (5)
Marketing	2 (5)
Business ethics	1 (3)
Organizational behavior	1 (3)
Legal issues	1 (3)

^a^One paper can include more than one discipline; therefore, the sum can be larger than 100%.

### Analysis Based on Reason for Digitalization

The digitalization of the health care system is discussed in just over two-thirds (n=26, 68%) of all papers (Table 4). This comes as no surprise, since increasing the efficiency and effectiveness of health care today seems to be the primary focus of the majority of stakeholders [17]. Transforming business models through digitalization is much more radical and challenging, which may explain the comparatively low number of articles addressing this (n=16, 42%).

**Table 4:** Number of papers by the reason for digitalization (N=38)^a^.

Reason for digitalization	Number of papers, n (%)
Operational process	26 (68)
Business model	16 (42)

^a^One paper can include several dimensions; therefore, the sum can be larger than 100%.

### Analysis Based on Content Domain

The vast majority of the papers (n=30, 79%) deal with different issues related to eHealth (Table 5). This is consistent with a survey published by the World Health Organization [18], which revealed that 58% of its member countries already have an eHealth strategy. Arak and Wójcik [19] held that eHealth is the key to addressing the challenges of modern health care systems. Electronic health records are a very popular subtopic of the eHealth papers reviewed (n=11, 29%).

The second most frequently mentioned dimension is data health (n=12, 32%). The majority of the papers in this category (n=7, 18%) deal with methods to analyze data. This digital health dimension is relatively close to the next dimension—overarching challenges (n=7, 18%)—where the focus is on challenges related to the implementation and use of different technologies found in practice. The biggest issue, by far, is personal data privacy. Both dimensions being so close to each other is not unusual, since data analysis always touches on security issues as well.

The dimensions occurring least frequently in the literature reviewed are trend health (n=5, 13%) and tech health (n=3, 8%). The latter is in line with our expectations for 2 reasons. First, we focused on management literature, and highly technical papers—where the hardware itself plays a major role—are unlikely to be published in business-oriented journals. Second, many of the technologies within the domain, such as 3D printing or robotics, are not yet widely spread in a health care context. More remarkable, however, is the low number of just 5 papers for trend health–related publications. From a business perspective, lifestyle solutions can be significant revenue drivers, as seen in the success of activity tracking devices. A possible explanation for this low count might be the bias of researchers to focus their activities on more ‘serious’ digital health aspects closer to conventional medicine.

**Figure 2:**
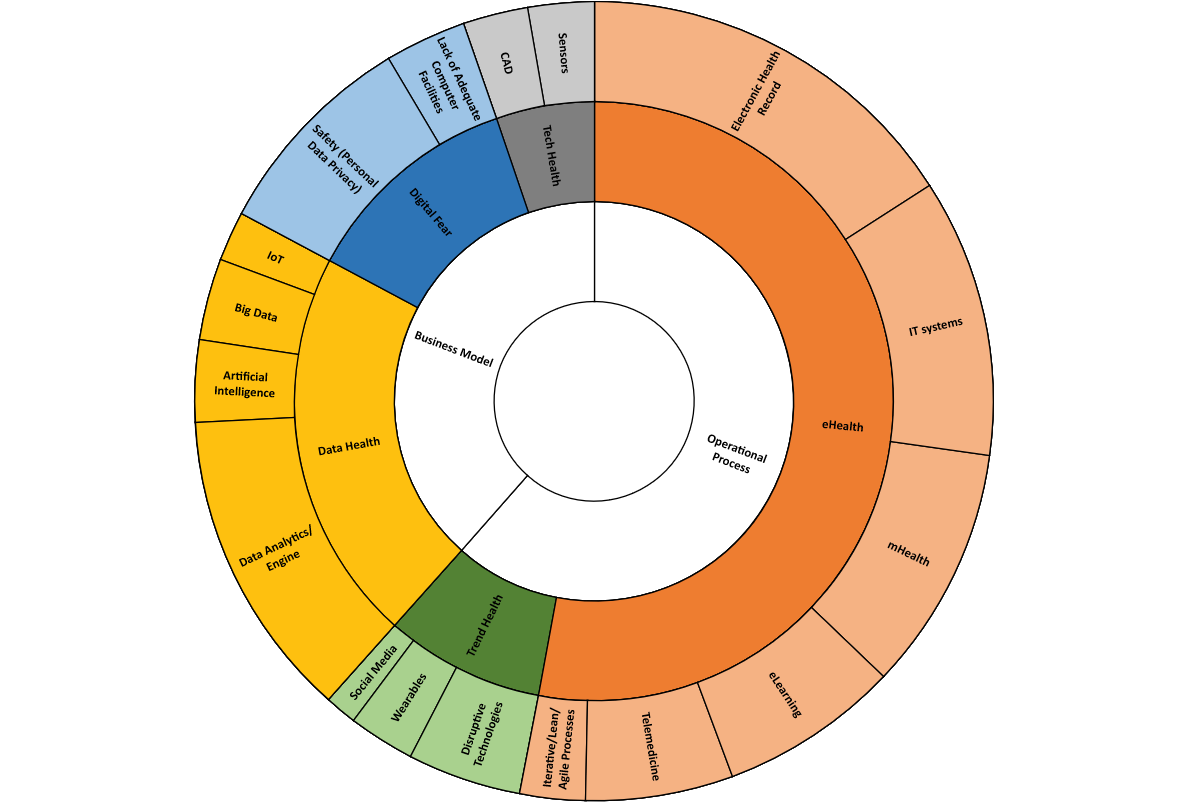
Distribution of reviewed publications. CAD: computer-aided design; IoT: internet of things; IT: information technology.

Figure 2 is a summary graphical representation of the content findings from our digital health literature search. The innermost (white) layer of the circle represents the ‘why’ and shows that operational processes are the main reason for digital transformation in the papers analyzed. The second layer represents the ‘where’ and shows clearly that eHealth is addressed most often in scientific articles. The outermost layer illustrates the ‘what’; it shows that electronic health records and IT systems are the topics mentioned most frequently.

**Table 5:** Distribution of publications by content domain (N=38)^a^.

Content domain	Total papers	Conceptual studies	Case-based studies
eHealth (exchange of data)	30 (79)	16 (42)	14 (37)
Data health (software)	12 (32)	5 (13)	7 (18)
Overarching challenges	7 (18)	2 (5)	5 (13)
Trend health (lifestyle)	5 (13)	1 (3)	4 (5)
Tech health (hardware)	3 (8)	1 (3)	2 (5)

^a^One paper can be part of several dimensions, so the sum can be larger than 100%.

## Discussion

### Main Findings

This literature review provides an overview of digital health literature from a management perspective and uncovers research gaps. In general, it shows that digitalization in the health care sector is still in its infancy, and therefore, there are still significant knowledge gaps concerning health care management. In the following section, we present the 3 important avenues for future research derived from our literature review.

The first finding regarding the ‘management discipline’ is that most papers analyzed focus on the IT domain and its technological aspects. Understanding technology is certainly key, as new IT solutions can enable management practices that were not previously possible. However, the technological perspective alone is not sufficient, as we strongly agree with Henriette et al [6] that “digital transformation is more than just a technological shift.” The implementation of digital health solutions could have major implications in many areas, such as for the senior management of a hospital [20]. For them, an innovation that would create the ability to track material and persons with sensors in real time could revolutionize the daily operational processes in larger hospitals. Consequently, we need more initiatives in which technology and management experts work together through all the development and implementation phases. True innovations can only happen when digital health technology is successfully integrated into daily operations and day-to-day management processes. Our first research proposition is hence to take more into consideration the implications for the management and leading of health care organizations, as follows:

- Future digital health research should not merely focus on the technology aspects. Instead, it should have a more holistic approach and further study the implications the technology has for the strategic and operational management of health care organizations.

The second research proposition refers to ‘content domains.’ We identified a strong focus on eHealth topics, as this has been the domain with the longest implementation history (eg, telemedicine was introduced to Australia back in the 1980s). Nevertheless, other fields could be of equal or greater importance when their implementation becomes more widespread. We base these expectations on the many publications forecasting a radical transformation of health care owing to different developing technologies (eg, in the field of big data, as in the review by Kruse et al [21]). Therefore, we advocate a more balanced examination of the 5 content domains presented in this paper, as follows:

- Explore the management perspective of all the different content dimensions of digital health in appropriate depth.

A final imbalance was found in the distribution of ‘research questions.’ An overwhelming number of papers create concepts for a possible digital health future. From a business perspective, these provide mostly anecdotal descriptions of the impact such solutions might have on the management of health care organizations. We suggest this is due to the relative youth of digital health in our current systems and a lack of widespread real-life application and experience. However, as more and more concepts become a reality, we would encourage the scientific community to take further steps. Future research should analyze in greater detail the real value of implementation. Practitioners and academics alike need more evidence regarding the expected outcome and impact of digital health on our health care organizations. Future research initiatives could, for example, examine the input and outcomes of digital health implementation by employing a quantitative study design. The quantification of the impact of digital health solutions on clinical outcomes has been the focus of many studies (eg, [22,23]). We encourage scholars to conduct methodologically similar pre-post studies, analyzing the effect on management process and business performance. A solid foundation is needed to result in meaningful recommendations for managers. Therefore, our last research avenue for exploration is the following:

- Broaden the research focus to include quantitative analysis of the impact of digital health on health care management and organizational design.

### Strengths

This study presents contributions to an underresearched area of digital health from a management viewpoint. Within the scope of our review, we examined the most relevant publications to access the impact of digital health on health care management. All of the available publications were published between 2010 and 2019, with more literature published from 2016 to 2019 emphasizing the growing interest in digital health. The findings of our systematic review are the first step toward giving health care practitioners an overview of digital health in the field of health care management and enabling them to handle the ongoing digital transformation in the best possible way. By unveiling the actual status of digitalization in the health care sector and showing significant knowledge gaps, we set the stage for further research on how best to support health care practitioners through the process of digital transformation. To achieve this in the best possible way, we conducted three research proposals for future research avenues based on the research gaps identified.

### Limitations

It is important to note some limitations associated with the study design. The researchers have a background in health care management and are based in Europe. We did not control for the geographical location of the papers analyzed, but our assumption is that most of the papers dealt with findings from North American and European organizations. A potential concern is therefore the generalization of the findings to other parts of the world. Furthermore, we focused on 3 specific databases as part of our systematic search. Therefore, it cannot be ruled out that the inclusion of further databases, such as Embase, might have yielded additional relevant publications. However, the studies published on Embase often coincide with those on PubMed, which was included in our search strategy. A final limitation is that no unpublished literature was reviewed or evaluated. However, due to the inclusion of various databases, we assume that the noninclusion of further publications is rather low. We therefore believe that with our approach, we have nevertheless achieved our aim to provide a first overview for practitioners.

### Conclusions

To our knowledge, this study is the first overview of the impact of digital health from the health care management viewpoint. This paper relies on a systematic literature review of both conceptual and case-based papers on digital health. As our main contribution, we have developed three research proposals for future research avenues based on the research gaps identified. The big question is not whether further developments in digital health will have an impact on our health care organizations but how prepared managers are to deal with these changes. We note that most publications are still concerned with possibilities, but as more and more of these possibilities become a reality, managers need to be able to be proactive and, what is more, shape their organizations accordingly. We believe this paper has created some insights for the digital health research community on how best to support health care practitioners through the process of digital transformation.

## Appendix

Multimedia Appendix 1 Literature review stages.

Multimedia Appendix 2 The specific inclusion criteria for each database.

Multimedia Appendix 3 All publications eventually included in this study.

## Abbreviations

IT: information technology
