# Supplementary figures and images for: The Management Perspective in Digital Health Literature: Systematic Review
Source: JMIR Mhealth Uhealth. 2022 Nov 10;10(11):e37624. doi: 10.2196/37624 (PMC9693713; doi:10.2196/37624)

**Multimedia Appendix 1. Literature review stages based on Tranfield et al. [7].**


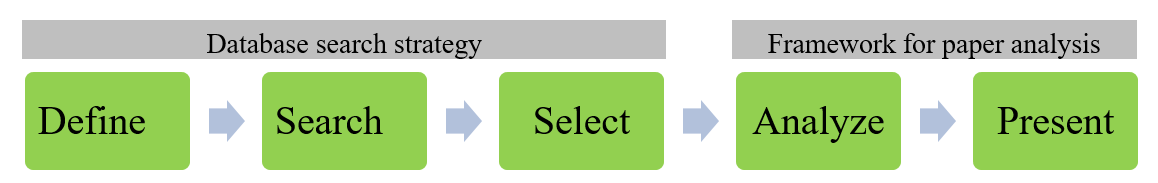

Supplement: Multimedia Appendix 1 [file mhealth_v10i11e37624_app1.docx]
