# Supplementary material for: The Management Perspective in Digital Health Literature: Systematic Review
Source: JMIR Mhealth Uhealth. 2022 Nov 10;10(11):e37624. doi: 10.2196/37624 (PMC9693713; doi:10.2196/37624)
Supplement: Multimedia Appendix 2 [file mhealth_v10i11e37624_app2.docx]

**Multimedia Appendix 2. The specific inclusion criteria for each database.**

| **Database** | **Search algorithm** | **Date** | **Language** | **Paper type** | **Paper type** | **Hits** |
| --- | --- | --- | --- | --- | --- | --- |
| ABI/Inform Global | ab(digital* AND health* AND manage*) | 2009-2019 | English | Full paper and  peer reviewed | Scientific  journals | 55 |
| Web of Science Core Collection | TITLE: (Digital*)  *AND* TITLE: (Health*) *AND* TOPIC: (Manage*) | 2000-2019 | English | Open access | Article | 68 |
| PubMed | (digital health*[Title]) AND manage* [all fields] | 10 years | English | Free full text | Humans | 23 |
| Total Hits | | | | | | 146 |
| Minus Duplicates | | | | | | 13 |
| **Net Hits** | | | | | | **133** |
