# Supplementary material for: The Management Perspective in Digital Health Literature: Systematic Review
Source: JMIR Mhealth Uhealth. 2022 Nov 10;10(11):e37624. doi: 10.2196/37624 (PMC9693713; doi:10.2196/37624)
Supplement: Multimedia Appendix 3 [file mhealth_v10i11e37624_app3.docx]

**Multimedia Appendix 3. All publications eventually included in this study.**

1. Abril-Gonzalez M, Portilla FA, Jaramillo-Mejia MC. Standard Health Level Seven for Odontological Digital Imaging. *Telemed e-Health*. 2017;23(1):66-73. doi:10.1089/tmj.2015.0251

2. Adams KT. Digital Health Technology Adoption Depends On Tech Comfort Level, Willingness to Pay. *Manag Care.* 2015;24(12):27-28.

3. Ahmadvand A, Gatchel R, Brownstein J, Nissen L. The Biopsychosocial-Digital Approach to Health and Disease: Call for a Paradigm Expansion. *J Med Internet Res.* 2018;20(5):e189. doi:10.2196/jmir.9732

4. Alshameri F, Hockenberry D, B. Doll R. The map is not the territory: the missing patient in the electronic medical record. *VINE*. 2014;44(4):548-557. doi:10.1108/VINE-03-2014 0024

5. Bezerra B, Alves E. PP156 New Information And Communication Technologies And Hospitals’ Design. *Int J Technol Assess Health Care*. 2017;33:141-142. doi:10.1017/S0266462317002951

6. Blaya JA, Fraser HSF, Holt B. E-Health Technologies Show Promise In Developing Countries. *Health Aff*. 2010;29(2):244-251. doi:10.1377/hlthaff.2009.0894

7. Bottles K MD, Kim J MD, MPH. Presentations Offer Insight on Social Media for Physicians. *Physician Executive*. 2013;39(3):92-93.

8. Cano I, Lluch-Ariet M, Gomez-Cabrero D, et al. Biomedical research in a Digital Health Framework. *J Transl Med.* 2014;12:S10. doi:10.1186/1479-5876-12-S2-S10

9. Chakravorty T, Jha K, Barthwal S. Digital Technologies as Enablers of Care-Quality and Performance: A Conceptual Review of Hospital Supply Chain Network. *IUP Journal of Supply Chain Management*. 2018;15(3):7-25.

10. Despotou G, Ryan M, Arvanitis TN, et al. A framework for synthesis of safety justification for digitally enabled healthcare services. *Digit Health.* 2017;3:UNSP 2055207617704271. doi:10.1177/2055207617704271

11. Faggini M, Cosimato S, Nota FD, Nota G. Pursuing Sustainability for Healthcare through Digital Platforms. *Sustainability*. 2019;11(1):165. doi:10.3390/su11010165

12. Falzon D, Timimi H, Kurosinski P, et al. Digital health for the End TB Strategy: developing priority products and making them work. *Eur Respir J*. 2016;48(1):29-45. doi:10.1183/13993003.00424-2016

13. Furberg RD, Ortiz AM, Zulkiewicz BA, Hudson JP, Taylor OM, Lewis MA. Supporting Tablet Configuration, Tracking, and Infection Control Practices in Digital Health Interventions: Study Protocol. *JMIR RES Protoc.* 2016;5(2):e136. doi:10.2196/resprot.5400

14. Garcia Rojo M, Morillo Castro A, Goncalves L. COST Action “EuroTelepath”: digital pathology integration in electronic health record, including primary care centres. *Diagn Pathol*. 2011;6:S6. doi:10.1186/1746-1596-6-S1-S6

15. Gbadegeshin SA. The Effect of Digitalization on the Commercialization Process of High-Technology Companies in the Life Sciences Industry. *Technol. Innov. Manag. Rev*. 2019;9(1):49-63.

16. Hanisch SE, Birner UW, Oberhauser C, Nowak D, Sabariego C. Development and Evaluation of Digital Game-Based Training for Managers to Promote Employee Mental Health and Reduce Mental Illness Stigma at Work: Quasi-Experimental Study of Program Effectiveness. *JMIR Ment Health*. 2017;4(3):UNSP e31. doi:10.2196/mental.7600

17. Huang F, Blaschke S, Lucas H. Beyond pilotitis: taking digital health interventions to the national level in China and Uganda. *Global Health*. 2017;13(1):49. doi:10.1186/s12992-017-0275-z

18. Huber C, Gärtner C. Digital Transformations in Healthcare Professionals’ Work: Dynamics of Autonomy, Control and Accountability **. *Management Revue*. 2018;29(2):139-161. doi:http://dx.doi.org/10.5771/0935-9915-2018-2-139

19. Konduri N, Aboagye-Nyame F, Mabirizi D, et al. Digital health technologies to support access to medicines and pharmaceutical services in the achievement of sustainable development goals. *Digit Health*. 2018;4:1-26. doi:10.1177/2055207618771407

20. Lerner JC, Robertson DC, Goldstein SM. Case Studies On Forecasting For Innovative Technologies: Frequent Revisions Improve Accuracy. *Health Aff.* 2015;34(2):311-318. doi:http://dx.doi.org/10.1377/h

21. Louise Hamilton A, Coldwell-Neilson J, Craig A. Development of an information management knowledge transfer framework for evidence-based occupational therapy: [1]. *VINE.* 2014;44(1):59-93.

22. Lupton D. Digital health now and in the future: Findings from a participatory design stakeholder workshop. *Digit Health*. 2017;3:UNSP 2055207617740018. doi:10.1177/2055207617740018

23. Malhotra S, Chakrabarti S, Shah R. A model for digital mental healthcare: Its usefulness and potential for service delivery in low- and middle-income countries. *Indian J Psychiatry.* 2019;61(1):27-36. doi:10.4103/psychiatry.IndianJPsychiatry_350_18

24. Michie S, Yardley L, West R, Patrick K, Greaves F. Developing and Evaluating Digital Interventions to Promote Behavior Change in Health and Health Care: Recommendations Resulting From an International Workshop. *J Med Internet Res*. 2017;19(6):e232. doi:10.2196/jmir.7126

25. Minor JM, Rickey LM, Bergenstal RM. Digital Health Care by In Silico Glycation of HbA1 Blood Cells. *J Diabetes Sci Technol*. 2017;11(5):975-979. doi:10.1177/1932296817700920

26. Parker DJ, Nuttall GH, Bray N, et al. A randomised controlled trial and cost-consequence analysis of traditional and digital foot orthoses supply chains in a National Health Service setting: application to feet at risk of diabetic plantar ulceration. *J Foot Ankle Res*. 2019;12:2. doi:10.1186/s13047-018-0311-0

27. Powell J, Wyatt J, van Velthoven MH. OP28 Health Apps: A Proposed Framework To Guide Clinical Risk Assessment. *Int J Technol Assess Health Care*. 2017;33:13-14. doi:10.1017/S0266462317001283

28. Ross J, Stevenson F, Dack C, et al. Developing an implementation strategy for a digital health intervention: an example in routine healthcare. *BMC Health Serv Res*. 2018;18(1):794. doi:10.1186/s12913-018-3615-7

29. Saillour-Glenisson F, Duhamel S, Fourneyron E, et al. Protocole of a controlled before-after evaluation of a national health information technology-based program to improve healthcare coordination and access to information. *BMC Health Serv Res*. 2017;17. doi:10.1186/s12913-017-2199-y

30. Serbanati LD, Ricci FL, Mercurio G, Vasilateanu A. Steps towards a digital health ecosystem. *J Biomed Inform*. 2011;44(4):621-636. doi:10.1016/j.jbi.2011.02.011

31. Sood MR, Toornstra A, Sereno MI, Boland M, Filaretti D, Sood A. A Digital App to Aid Detection, Monitoring, and Management of Dyslexia in Young Children (DIMMAND): Protocol for a Digital Health and Education Solution. *JMIR RES Protoc*. 2018;7(5):e135. doi:10.2196/resprot.9583

32. Towery H, Hough M. Digital and face-to-face advances and detractions to cure the personal and financial cost of misdiagnosis. *Strategic HR Review*. 2018;17(1):39-43.

33. Tsai A. An Integrated e-Learning Solution in Hospitals. *Journal of Global Business Issues*. 2010;4(2):85-93.

34. Urhuogo I, Vann V, Chandan HC. Global Information Systems Innovation: Healthcare Digital Records. *J. Bus. Stud. Q*. 2013;4(3):1-12.

35. Vezyridis P, Timmons S. On the adoption of personal health records: some problematic issues for patient empowerment. *Ethics Inf. Technol.* 2015;17(2):113-124. doi:10.1007/s10676-015-9365-x

36. Wallin AJ, Fuglsang L. Service innovations breaking institutionalized rules of health care. *J. Serv. Manag*. 2017;28(5):972-997. doi:10.1108/JOSM-04-2017-0090

37. Watsuji T. The Current State of Digital Healthcare towards Medical Application. *IEICE Trans Commun*. 2016;E99B(3):565-568. doi:10.1587/transcom.2015MII0002

38. Wysham NG, Abernethy AP, Cox CE. Setting the vision: applied patient-reported outcomes and smart, connected digital healthcare systems to improve patient-centered outcomes prediction in critical illness. *Curr Opin Crit Care*. 2014;20(5):566-572. doi:10.1097/MCC.0000000000000139
